# Association of total testosterone status with bone mineral density in adults aged 40–60 years

**DOI:** 10.1186/s13018-021-02714-w

**Published:** 2021-10-18

**Authors:** Nan Wang, Lixiang Wang, Chengcheng Huang

**Affiliations:** 1 Department of Orthopaedics, Zhejiang Chinese Medical University Affiliated Jiangnan Hospital, 152 Yucai Road, Chengxiang Street, Xiaoshan District, Hangzhou City, 311200 Zhejiang Province China; 2 Zhejiang Chinese Medical University, Hangzhou, 310053 Zhejiang China; 3 Department of Osteoporosis Care and Control, The First People’s Hospital of Xiaoshan District, Hangzhou, 311200 Zhejiang China

**Keywords:** Testosterone, Bone mineral density, Adults, NHANES, Cross-sectional study

## Abstract

**Objective:**

Evidence linking total testosterone and bone mineral density (BMD) in adults is very limited. According to our review of the literature, only a few reports have focused on the relationship between total testosterone and bone mineral density in adults. Therefore, the purpose of this study was to determine the relationship between total testosterone and total bone mineral density in adults aged 40–60 years.

**Methods:**

We used a cross-sectional study of a non-institutionalized U.S. population sample from the National Health and Nutrition Examination Survey. A weighted multivariate linear regression model was used to evaluate the relationship between total testosterone and total bone mineral density. Subgroup analyses were further performed.

**Results:**

In multiple regression models adjusted for potential confounders, total testosterone levels were inversely associated with total bone mineral density. However, in the sex-stratified subgroup analysis, the association between total testosterone levels and total bone mineral density was not significant in female adolescents. There was no negative association between total testosterone and total BMD among men, adults 40 to 60 years of age, and other racial/ethnic groups. There is a negative association between total testosterone and total bone mineral density when total testosterone concentration is greater than 500 ng/dL among Non-Hispanic black.

**Conclusion:**

Our statistical results show that the association between total testosterone levels and total bone mineral density varies by gender and race. Elevated total testosterone levels below 500 ng/dL have adverse effects on bone health. Total testosterone concentrations below 500 ng/dL may have no effect on bone health.

## Introduction

Osteoporosis is a common clinical disease. Studies have shown that after menopause, women have lower estrogen levels, lower bone density, and develop osteoporosis. Bone mineral density was positively correlated with estrogen levels in women. However, the relationship between male osteoporosis and hormone levels is often ignored, and a large number of patients do not receive timely diagnosis and treatment.

As age increases, the level of Testosterone in middle-aged men gradually decreases, leading to the occurrence of Testosterone deficiency. The traditional view is that male osteoporosis is similar to female osteoporosis, and sex hormone levels play an important role in regulating bone mass and metabolism. Men with hypogonadism have faster bone metabolism, an increased risk of osteoporosis and brittle fractures. Men with osteoporosis have lower testosterone levels.

Convincing experimental studies reported that bone mineral density seems to be influenced by testosterone levels and men's exercise [1]. The sex hormones deficit can predict the reduction of BMD in men [2]. A study population consisted of 2447 community-dwelling men in America showed that older men with total testosterone were more likely to be osteoporotic [3]. People with osteoporosis are more likely to have a total lack of testosterone. Men who are completely deficient in testosterone are more likely to lose bone rapidly. It may be clinically necessary to detect BMD in elderly men with sex hormone deficiency [4, 5].Moreover, increasing evidence supported that there is a strong link between testosterone concentration and bone mineral density [6, 7].

Recent epidemiological studies reported a positive association between total testosterone and BMD in the middle-aged and older men [4, 8]. Until now, evidence linking total testosterone and BMD in men is very limited [9, 10]. Osteoporosis in middle-aged men varies from country to country, depending on age, sex, ethnicity and region.In addition, testosterone concentration is affected by age, sex, body composition, and other factors that influence BMD [11].Therefore, we conducted a larger cross-sectional study based on the national population to determine the association between testosterone and BMD in men aged 40 to 60 years.

## Materials and methods

### Statement of ethics

This study was approved by the ethics review board of the National Center for Health Statistics, and written consent was obtained from each participant.

### Study population

The data analyzed came from the National Health and Nutrition Examination Survey (NHANES) (2011–2016), which is a stratified, multi-stage probability sample of the uninstitutionalized U.S. population. These cross-sectional surveys are conducted by the National Center for Health Statistics (NCHS). A detailed approach to NHANES is available at http://www.cdc.gov/nchs/nhanes/.

### Variables

Study subjects were limited to participants aged 40–60 years (n = 6005). The NCHS Ethics Review Board approved the actions of NHANES and obtained the written informed consent of all participants.

### Study variables

The main variables in this study were total testosterone (independent variable) and total BMD (dependent variable).

This is a validated isotope dilution liquid chromatography tandem mass spectrometry (ID-LC–MS/MS) method for routine quantitation of serum total testosterone based on the National Institute for Standards and Technology’s (NIST) reference method. Total BMD was measured by dual-energy X-ray absorptiometry.

In addition, the following covariates were included: age, sex, race/ethnicity, level of education, income-poverty ratio, total protein, serum phosphorus, and serum calcium. Detailed information about the data measurement process is available at http://www.cdc.gov/nchs/nhanes/.

### Statistical analyses

All estimates were calculated accounting for NHANES sample weights. Weighted multiple regression analysis was applied to estimate the independent relationship between total testosterone and total BMD. Weighted generalized additive models and smooth curve fittings were employed to address the non-linearity of total BMD and total BMD in the subgroup analyses.

Categorical variables were expressed as frequency or percentage. Continuous variables were expressed as means ± standard deviation. Weighted linear regression models (continuous variables) and weighted chi-square tests (categorical variables) were performed to calculate differences between different groups. *P* < 0.05 was considered statistically significant. All analyses were performed with Empower software (http://www.empowerstats.com; X&Y solutions, Inc., Boston MA) and R version 3.4.3 (http://www.R-project.org, The R Foundation).

## Results

**Fig. 1:**
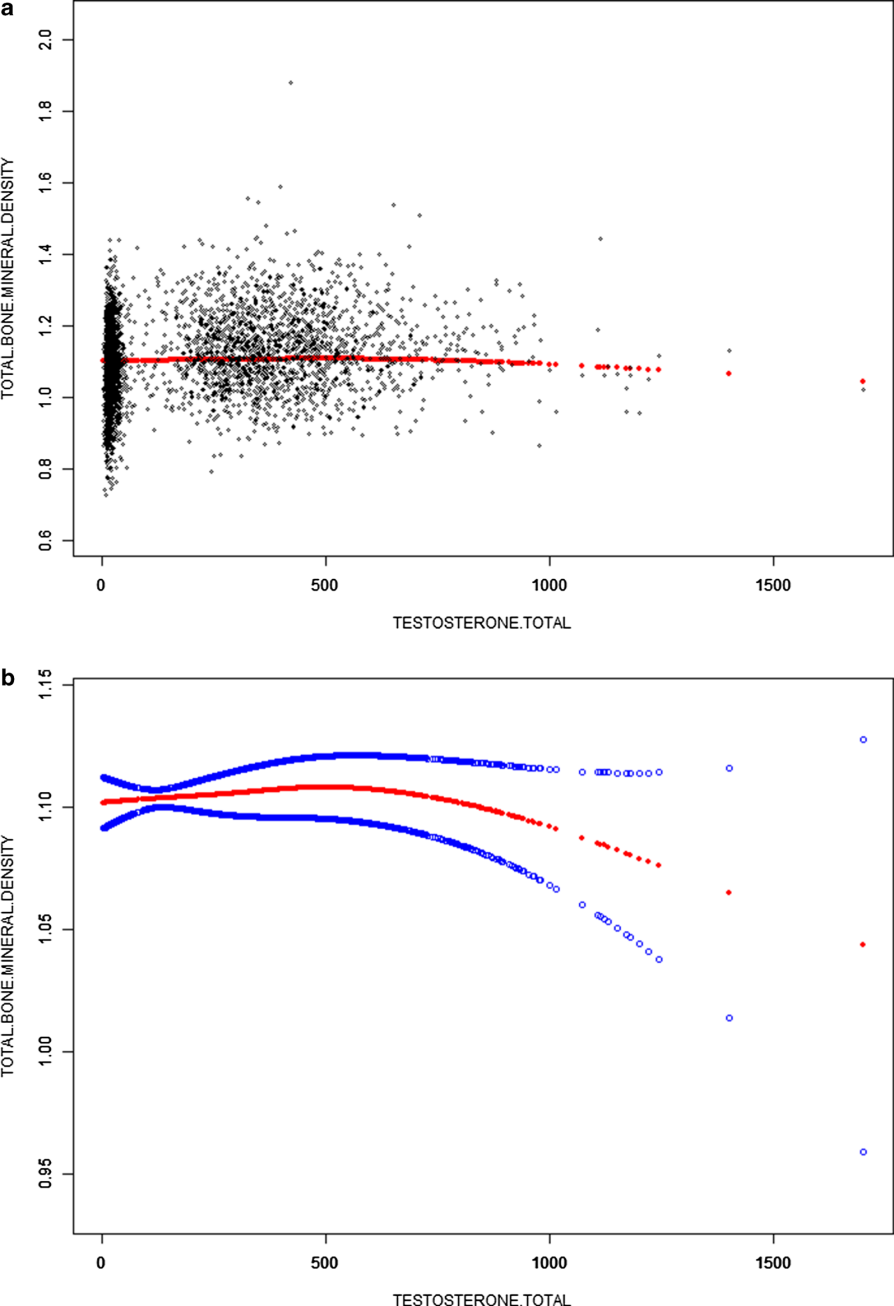
The association between total testosterone and total BMD. **a** Each black point represents a sample. **b** Solid rad line represents the smooth curve fit between variables. Blue bands represent the 95% of confidence interval from the fit. Age, sex, race/ethnicity, education, income poverty ratio, total protein, serum phosphorus, and serum calcium were adjusted

**Fig. 2:**
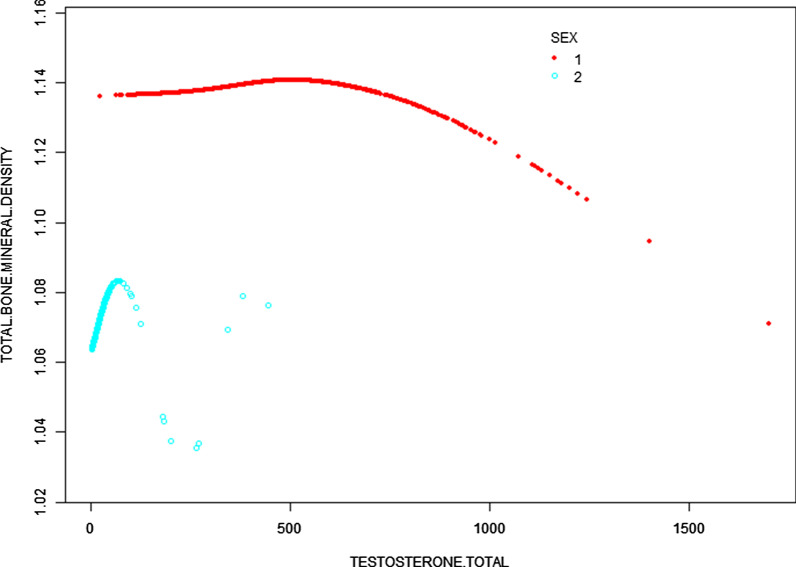
The association between total testosterone and total BMD stratified by sex. Age, race/ethnicity, education, income poverty ratio, total protein, serum phosphorus, and serum calcium were adjusted

**Fig. 3:**
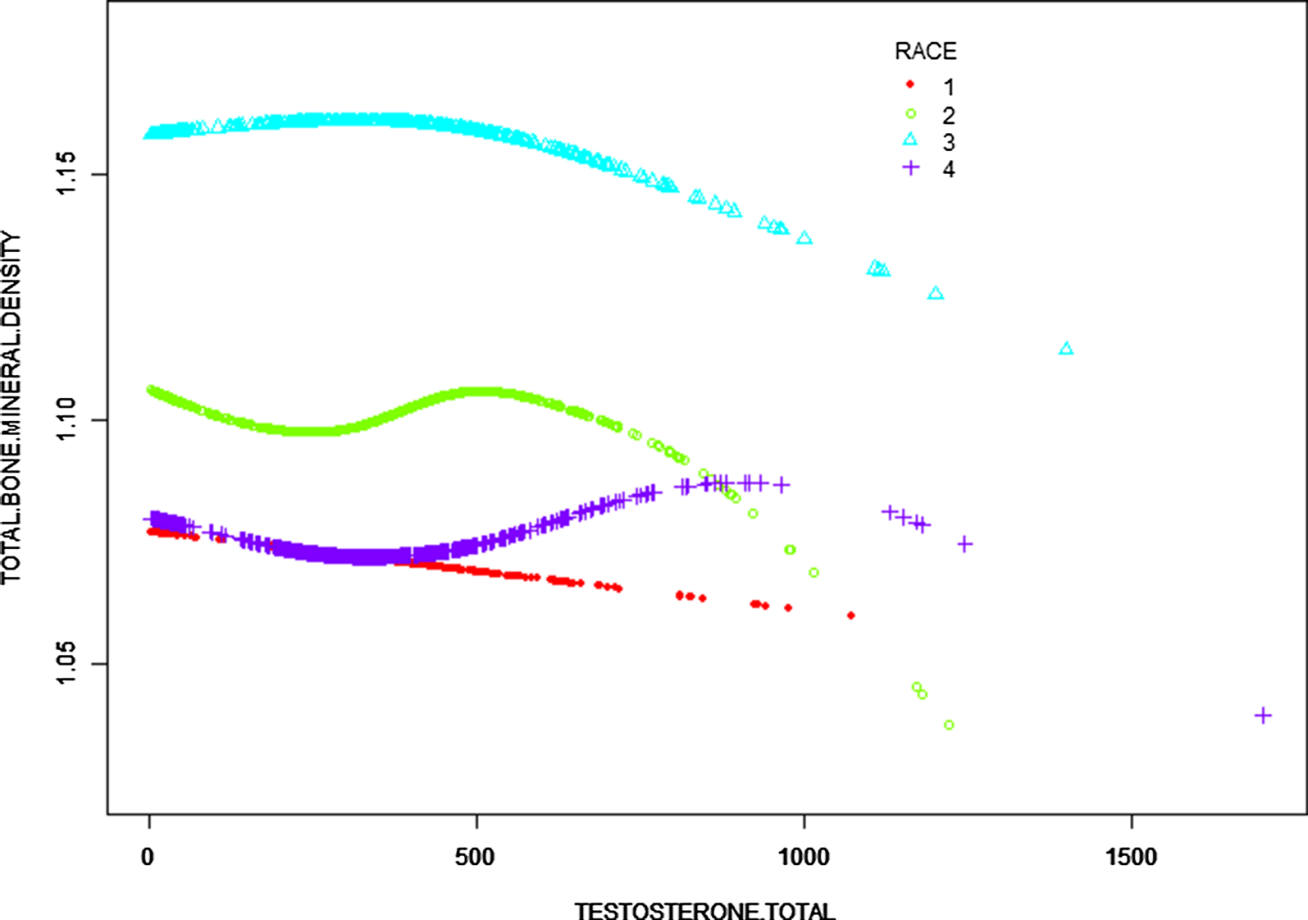
The association between total testosterone and total BMD stratified by race/ethnicity. Age, sex, education, income poverty ratio, total protein, serum phosphorus, and serum calcium were adjusted

Table 1 shows the description of weighted sociodemographic and medical characteristics of the participants. A total of 6005 participants were included in this study. Of these participants, 47.34% were male, 52.66% were female, 13.96% were Non-Hispanic white, 34.12% were Non-Hispanic black, 23.93% were Mexican Americans, and 27.99% were other race. Total testosterone (quartiles, Q1–Q4) were significantly different groups, including age, sex, race/ethnicity, income-poverty ratio, total protein, serum phosphorus, serum calcium, and total BMD (Table 2, Figs. 1, 2, 3).

**Table 1:** Weighted characteristics of the study population based on total testosterone quartiles

Testosterone total (ng/dL)	Total	Q1	Q2	Q3	Q4	*P* value
Age (years)	49.9 ± 6.1	50.3 ± 5.8	49.6 ± 6.0	50.0 ± 6.0	50.2 ± 6.2	0.0148
Sex (%)						< 0.0001
Male	47.3	0.1	0.0	88.6	99.8	
Female	52.7	99.9	100	11.4	0.2	
Race/ethnicity (%)						0.0284
Non-Hispanic white	14.0	9.7	6.9	7.5	8.4	
Non-Hispanic black	34.1	63.0	68.2	67.8	68.2	
Mexican American	23.9	12.2	12.2	10.5	10.1	
Other race/ethnicity	28.0	15.1	12.7	14.2	13.4	
Level of education (%)						< 0.0001
Less than high school	23.2	14.1	13.4	13.5	18.0	
High school	21.5	18.2	18.6	22.3	22.5	
More than high school	55.3	67.7	68.0	64.3	59.5	
Income to poverty ratio	2.7 ± 1.7	3.2 ± 1.7	3.2 ± 1.7	3.4 ± 1.6	3.2 ± 1.7	0.0106
Total protein (mg/dL)	71.5 ± 4.7	70.3 ± 4.6	70.4 ± 4.4	70.8 ± 4.5	70.9 ± 4.4	0.0013
Serum calcium (mg/dL)	9.4 ± 0.4	9.4 ± 0.4	9.3 ± 0.4	9.4 ± 0.4	9.4 ± 0.3	0.1272
Serum phosphorus (mg/dL)	3.7 ± 0.6	3.9 ± 0.5	3.8 ± 0.5	3.7 ± 0.6	3.6 ± 0.6	< 0.0001
Total BMD (g/cm^2^)	1.1 ± 0.1	1.1 ± 0.1	1.1 ± 0.1	1.1 ± 0.1	1.1 ± 0.1	< 0.0001

Mean ± SD for continuous variables: the *P* value was calculated by the weighted linear regression model. (%) for categorical variables: the *P* value was calculated by the weighted chi-square test. BMD, bone mineral density

**Table 2:** The association between total testosterone (ng/dL) and total bone mineral density (g/cm^2^)

	Model 1		Model 2		Model 3	
	β (95% CI)	*P* value	β (95% CI)	*P* value	β (95% CI)	*P* value
Total testosterone (ng/dL)	0.0001 (0.0001, 0.0001)	< 0.000001	0.0000 (− 0.0000, 0.0000)	0.907372	0.0001 (0.0001, 0.0002)	< 0.000001
Total testosterone categories						
Q1	0		0		0	
Q2	0.0067 (− 0.0026, 0.0160)	0.159252	0.0034 (− 0.0055, 0.0123)	0.449622	0.0057 (− 0.0035, 0.0150)	0.223303
Q3	0.0633 (− 0.0540, 0.0726)	< 0.000001	0.0023 (− 0.0171, 0.0217)	0.815594	0.0044 (− 0.0154, 0.0242)	0.665964
Q4	0.0775 (0.0682, 0.0867)	< 0.000001	0.0104 (− 0.0108, 0.0315)	0.335823	0.0155 (− 0.0063, 0.0372)	0.162864
Subgroup analysis stratified by sex						
Men	0.0000 (− 0.0000, 0.0000)	0.719289	0.0000 (− 0.0000, 0.0000)	0.864406	0.0000 (− 0.0000, 0.0000)	0.36741
Women	− 0.0001 (− 0.0002, 0.0001)	0.492458	− 0.0001 (− 0.0002, 0.0001)	0.270182	− 0.0001 (− 0.0003, 0.0000)	0.151631
Subgroup analysis stratified by race/ethnicity						
Non-Hispanic white	0.0001 (0.0001, 0.0001)	< 0.000001	− 0.0001 (− 0.0001, 0.0000)	0.062793	− 0.0001 (− 0.0001, 0.0000)	0.324209
Non-Hispanic black	0.0001 (0.0001, 0.0002)	< 0.000001	0.0000 (− 0.0000, 0.0001)	0.521033	0.0000 (− 0.0000, 0.0001)	0.303087
Mexican American	0.0001 (0.0001, 0.0001)	< 0.000001	− 0.0001 (− 0.0001, 0.0000)	0.05225	− 0.0000 (− 0.0001, 0.0000)	0.155034
Other race/ethnicity	0.0001 (0.0001, 0.0002)	< 0.000001	0.0000 (− 0.0000, 0.0001)	0.193526	0.0000 (− 0.0000, 0.0001)	0.315806

Model 1: no covariates were adjusted. Model 2: age, sex, and race/ethnicity were adjusted. Model 3: age, sex, race/ethnicity, education, income poverty ratio, total protein, serum phosphorus and serum calcium were adjusted. In the subgroup analysis stratified by sex and race/ethnicity, the model is not adjusted for sex and race/ethnicity, respectively

## Discussion

The main findings of our study were as follows: First is the association between total testosterone levels and total bone mineral density was not significant in female adolescents. Second, there was no negative association between total testosterone and total BMD among men, adults 40 to 60 years of age, and other racial/ethnic groups when total testosterone concentration is less than 500 ng/dL. Third, there is a negative association between total testosterone and total bone mineral density when total testosterone concentration is greater than 500 ng/dL among Non-Hispanic white, Non-Hispanic black and Mexican Americans .

Low total bone mineral density is associated with an increased risk of osteoporotic fracture. The association between total testosterone and BMD received extensive research and attention over the years. In a study of 1070 Korean men, J shin et al. identified that both total testosterone and free testosterone were positively associated with BMD and that genetic effects were significant on the association between testosterone and BMD [12]. By contrast, In a study of factors associated with bone mineral density in young male distance runners, resistance training was associated with higher bone mineral density in young adult male distance runners, independent of physiological factors [9]. A study of 199 men with osteoporosis or loss of bone mass found that sex hormone deficiency predicted loss of bone mineral density, with estradiol and testosterone most significantly associated [13]. In a study of 399 men, more than a third of men younger than 50 years with testosterone deficiency and infertility or sexual dysfunction had decreased bone mineral density. Testosterone treatment increased bone mineral density. After testosterone treatment, spinal bone density increased significantly, improving lumbar osteoporosis in men. In this 2-year prospective open-air study, 50 men aged 50 to 65 years who took 50 mg of testosterone gel daily for 12 months significantly improved bone density in the lumbar spine and hip, and improved symptoms of osteoporosis pain [14].

More and more attention is paid to the relationship between total testosterone and markers of bone metabolism. Some scholars believe that the identification of the new determinants of bone mineral density loss in osteoporosis is a field in constant development [15]. Some researchers hope to find a molecular link between total testosterone and osteoblasts and osteoclasts to explain how total testosterone can affect bone mineral density. Researchers used data from 1338 men (25–86 years) in the population-based epidemiological Study of Health in Pomerania for a study [16]. The key finding of the study is the positive association between total testosterone and osteocalcin. Another study examined serum and urinary biochemical parameters of 43 healthy men aged 20–80 years. Univariate analysis of static bone morphometric parameters and biochemical parameters showed that the surface of osteoclasts was significantly correlated with serum total testosterone, indicating that testosterone plays an important role in the pathogenesis of osteoporosis [17].

In this study, we analyzed a representative sample of multiracial populations to better generalize the U.S. population. In addition, such a large sample size allowed us to conduct further subgroup analysis. This is the main advantage of this study. However, these limitations are worth noting. First, because of the cross-sectional design of this study, it is difficult to determine whether there is a causal relationship between total testosterone and total bone mineral density. Second, other confounding factors not included in this study may have influenced the results. For example, men's total testosterone levels are biologically higher than women's. Therefore, differences in sex hormones during adolescent development in adults aged 40–60 years may be a potential confounding factor to be considered. Therefore, it is necessary to further clarify the role of total testosterone in bone metabolism and conduct longitudinal follow-up studies with large sample size.

In summary, total testosterone level and total BMD differed by sex and race. For American men, the increased total testosterone level would have an adverse effect on bone health with high total testosterone levels (> 500 ng/dL), but may have no effect on bone health when total testosterone concentration is lower than 500 ng/dL.

## Abbreviations

BMD: Bone mineral density
BMI: Body mass index
NHANES: National Health and Nutrition Examination Survey
